# A method for estimating intracellular ion concentration using optical nanosensors and ratiometric imaging

**DOI:** 10.1038/s41598-017-11162-8

**Published:** 2017-09-07

**Authors:** Guoxin Rong, Eric H. Kim, Kira E. Poskanzer, Heather A. Clark

**Affiliations:** 1 Department of Bioengineering, Northeastern University, Boston, MA 02115 United States; 2 Department of Biochemistry & Biophysics and Kavli Institute for Fundamental Neuroscience, University of California, San Francisco, San Francisco, CA 94143 United States; 3 Department of Chemistry and Chemical Biology, Northeastern University, Boston, MA 02115 United States

## Abstract

Optical nanoparticle (NP)-based sensors have been widely implemented as tools for detection of targeted ions and biomolecules. The NP sensing platform offer a modular design that can incorporate different sensing components for greater target specificity and the ability to tune the dynamic range, as well as encapsulation of multiple dyes to generate a ratiometric signal with varying spectra. Despite these advantages, demonstrating quantitative ion imaging for intracellular measurement still possess a major challenge. Here, we describe fundamentals that enable intracellular validation of this approach using ion-selective nanosensors for investigating calcium (Ca^2+^) as a model ion. While conventional indicators can improve individual aspects of indicator performance such as Kd, wavelength, and ratiometric measurements, the use of NP sensors can achieve combined benefits of addressing these issues simultaneously. The nanosensor incorporates highly calcium-selective ionophores and two fluorescence indicators that act as signal transducers to facilitate quantitative ratiometric imaging. For intracellular Ca^2+^ application, the sensors are fine-tuned to physiological sensing range, and live-cell imaging and quantification are demonstrated in HeLa cells loaded with nanosensors and their responsiveness to carbachol-evoked store release (~400 nM). The current nanosensor design thus provides a promising sensing platform for real-time detection and optical determination of intracellular ions.

## Introduction

Optical imaging and fluorescent probes provide key tools for exploring the spatiotemporal dynamics of ions in living systems. In particular, real-time detection of ion dynamics in the cellular environment requires designing optical probes with high target specificity and sensitivity. Major progress has been achieved with the advent of fluorescent calcium (Ca^2+^) indicators^1, 2^, luminescent metal-complexes^3, 4^, quantum dots^5, 6^, and genetically encoded indicators^7, 8^ which continue to provide powerful tools for the imaging of ions. However, when applied to intracellular measurement, accurate determination of the intracellular concentration with these indicators can be complicated by a non-optimal dissociation constant (Kd), low signal, undesired compartmentalization, and interference with cellular components potentially leading to variability and inaccurate measurements. A number of fluorophores are commercially available to achieve quantitative ion imaging as demonstrated by single wavelength dyes in Ca^2+^ recordings^9, 10^, as well as other approaches including fluorescence resonance energy transfer (FRET)-based sensors^11, 12^. The accuracy of optical quantification is often improved by ratiometric methods which eliminates interference caused by fluctuations in excitation intensity, fluorophore concentration, and light scattering due to specimen thickness. Traditionally, quantitative Ca^2+^ imaging has been achieved with ratiometric indicator dyes such as Fura-2^2, 13^ and Indo-1^14^, and fluorescence lifetime imaging (FLIM) methods^15^. However, ratiometric imaging in practice is limited to only a few indicators in the UV spectrum associated with excitation light-induced phototoxicity, higher tissue scattering, and most importantly, the requirement of structural modification to alter its physiochemical properties such as the sensing range for desired biological applications.

Nanoparticle (NP)-based sensors^16–22^ provides an efficient and promising approach for developing robust imaging probes for quantitative ion detection. For example, while molecular probes can improve individual aspects of indicator performance such as Kd, wavelength, and ratiometric measurements, the use of NP-based sensors can achieve combined benefits of addressing these issues simultaneously. By incorporating multiple agents in the NP matrix, the advantages of these classes of sensors include: (i) brighter and photostable probes by integrating a high number of dyes; (ii) ability to fine-tune the sensor dynamic range; (iii) greater selectivity and range of analyte detection; (iv) adjustable emission spectra and ratiometric response; (v) reduced effects from light-induced cell damage^23^ by integrating longer wavelength dyes; and (vi) low cytoplasmic mobility^24–27^ to prevent dye diffusion, and protection from intracellular organelle sequestration^28, 29^. Indeed, the recent advances in NP-based sensors from various nanomaterials have been widely implemented in cellular tracking and imaging applications^16–22^. For example, there are several nano- and micro-particle sensors for detection of ions reported for sodium^30–32^, potassium^33–37^, calcium^38, 39^, magnesium^40, 41^, zinc^42^, silver^43^, and chloride^44^. Other efforts include nanosensors termed PEBBLEs (probes encapsulated by biologically localized embedding) developed by Kopelman and co-workers^28, 45^. These sensors incorporate molecular indicators such as rhod-2 into a polymeric matrix, however, the flexibility to tune the dynamic range remains challenging with this mechanism^46^. While the development of NP-based sensors has been an increasingly growing field of research, the application for measurement of intracellular ions, and detailed procedure for quantitative ratiometric ion imaging has not been fully established in these sensors.

To address this issue, we present a ratiometric sensor termed calcium-optode nanosensor (opCaNS) for quantitative fluorescence imaging of ions in living cells, and establish a procedure for estimating ionic concentration using ratiometric fluorescent signal. Calcium is selected as a model target ion to reliably assess our sensor characteristics to the extensive work established on the local control of Ca^2+^ signaling, as widely exemplified by cellular processes ranging from gene expression, synaptic transmission, hormone secretion to cell survival and death^47–50^.

In the nanosensor design, the ion-selective optodes (ISOs)^21, 46, 51^, an optical counterpart to ion-selective electrodes (ISEs), were applied and miniaturized to nanoscale for intracellular investigation. In the ISO sensing scheme, three reagents are embedded within the NP matrix: a highly selective, optically silent ion carrier (ionophore)^21, 46, 51^ that is capable of reversibly binding ions; a fluorescent pH indicator dye (chromoionophore) which acts as a reporter to yield a fluorescence readout based on its protonation degree; and a lipophilic charge-carrying molecule (ion exchanger) to maintain electroneutrality within the NP. Hence, ion-specific optical change is facilitated by binding of calcium ions by the ionophores, which causes displacement of protons of the pH-indicator with a quantifiable fluorescence signal. Furthermore, we have incorporated a second pH-indicator [octadecyl rhodamine (R18)] and term it as reference dye, whereby fluorescence intensity moves in opposite direction to pH changes in comparison to the chromoionophore to achieve a highly sensitive sensor for ratiometric measurement of intracellular Ca^2+^. Most importantly, the sensing range of the probe is easily modified and tuned by changing the ratio of components, which provides flexibility to the user for specific imaging applications.

Overall, the opCaNS provides a sensing platform suitable for intracellular Ca^2+^ measurement using ratiometric approach with sensor characteristics tuned to exhibit physiological sensing range, high selectivity, fast response time, and a reversible profile. Herein, we demonstrate the performance of this class of ion-selective nanosensors for cellular applications, achieving real-time detection and quantitative ion measurements by live-cell imaging for the first time.

## Results

### Principle and characterization of ratiometric nanosensor

**Figure 1:**
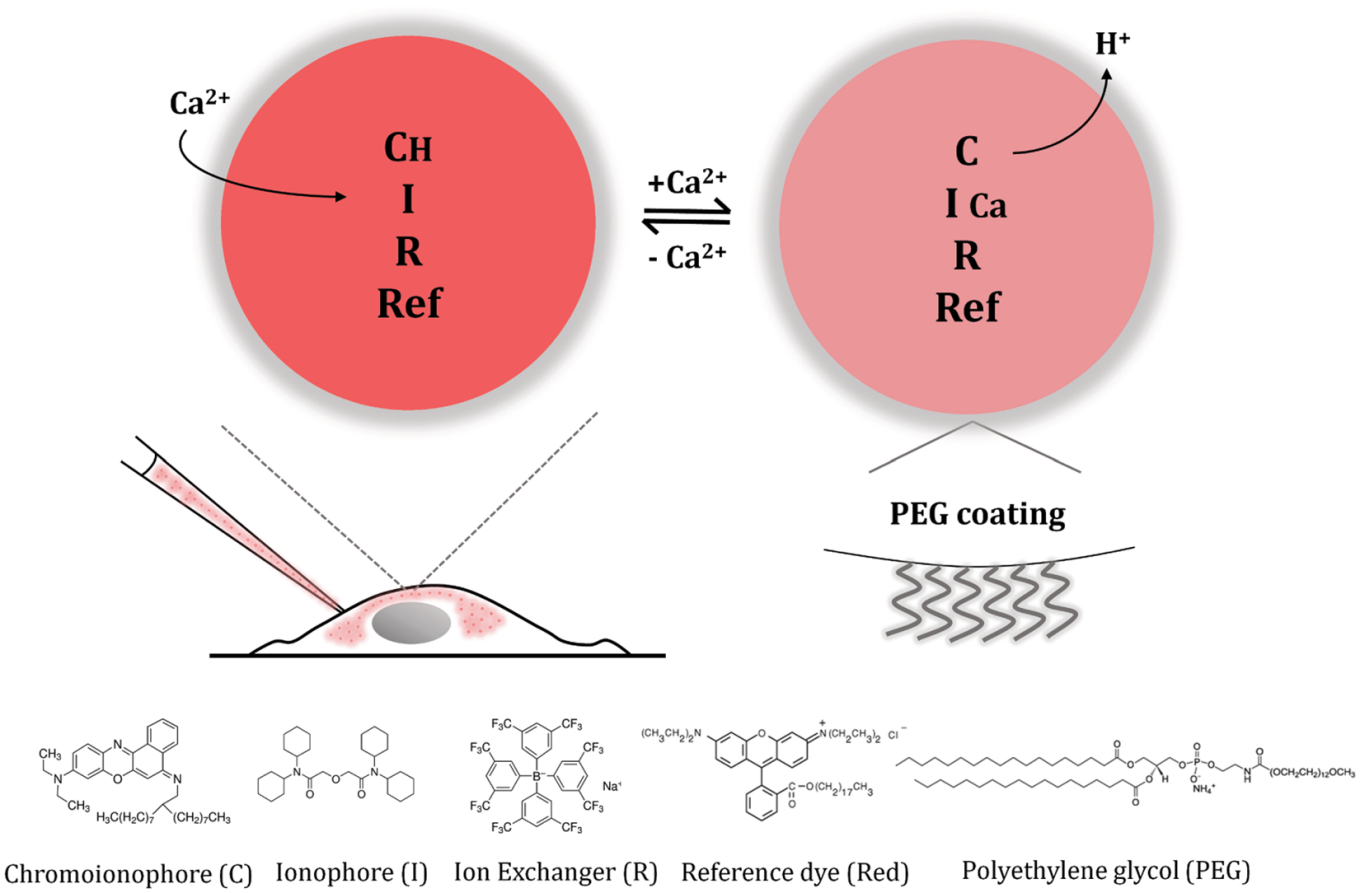
Calcium nanosensor working principle. The sensing components (chromoionophore, C; ionophore, I; ion exchanger, R) embedded in an organic hydrophobic sensor matrix. The recognition of target ion by selective ionophore-mediated recruitment results in an optical signal output as determined by the protonation degree of the chromoionophore. The overall electroneutrality of the sensor is maintained by ion exchanger. Ratiometric responses are obtained by incorporation of additional reference dye (Ref). The surface is covered with polyethylene glycol (PEG)-lipid molecules to offer particle stabilization. On the bottom are chemical structures and abbreviations of the main sensor components used in this work.

**Figure 2:**
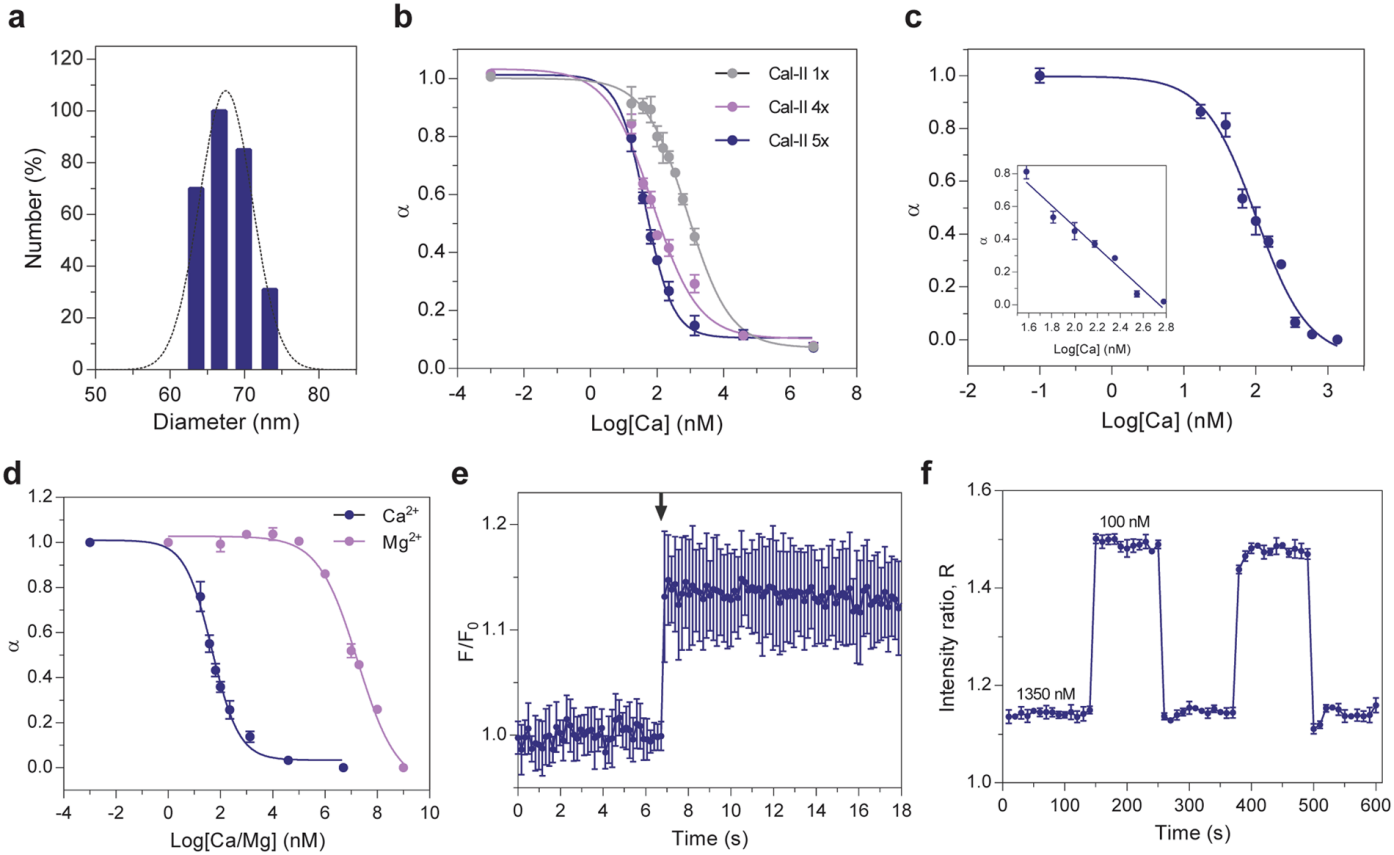
Sensor characterization and calibration. (**a**) Size distribution of nanosensors as measured by dynamic light scattering show an effective average diameter of 67 nm. (**b,c**) Calibration of the calcium nanosensors performed in solution. (**b**) Sensor response can be fine-tuned by altering amounts of sensing components. (**c**) For intracellular applications, the sensor response is tuned to a characteristic Ca^2+^ concentration derived from Hill fit (defined here as, EC_50_) at nanomolar range (105 ± 19 nM), with a corresponding linear range between 38 to 600 nM (inset). (**d**) Characterization of nanosensors in response to Ca^2+^ (blue) demonstrate 4 orders of magnitude selectivity over Mg^2+^ (purple). **(e)** Two-photon Ca^2+^ uncaging experiment using opCaNS. Nanosensors (*n* ﻿= 1﻿0) respond instantaneously to elevations of local Ca^2+^ levels by DMNP uncaging, as indicated by an increase of fluorescence levels from the base line (arrow). (**f**) Reversible response over repeated cycles between exchanged Ca^2+^ concentrations (100 nM and 1350 nM) characterized by confocal microscopy (*n﻿* = 3). Measurements are taken in triplicate from three individual sets of nanosensors (*n* = 9), unless specified otherwise. Data is shown as mean ± standard deviation (SD).

To achieve nanosensors for real-time detection and measurement of intracellular free calcium ions ([Ca^2+^]_i_), we applied ISOs as the principle sensing scheme, and further developed into miniaturized nanoscale sensors for ratiometric fluorescence imaging. In this sensing platform, the nanosensors are fabricated by co-immobilizing hydrophobic optode components comprised of calcium ionophore (I), a fluorescent chromoionophore (C), ion exchanger (R), and reference fluorophore (Ref) within a biocompatible plasticizer nano-emulsion matrix, and stabilized by a biocompatible polyethylene glycol (PEG)-lipid as surfactant (Fig. 1). The hydrodynamic diameter of nanosensors measured 67 nm (polydispersity index 0.16) (Fig. 2a) with zeta-potential of −29.9 ± 1.4 mV, indicating nanoparticles that are stable in aqueous solution. Mechanistically, the highly selective calcium ionophore acts as a recognition element that extracts Ca^2+^ into the sensor, which causes the pH-sensitive fluorophore (chromoionophore) to deprotonate to maintain charge neutrality within the sensor with an accompanying change in fluorescence. In this scheme, the opCaNS respond to Ca^2+^ in accordance with optode function (Eqs 1–3)^21, 46, 52^. The ion-exchange optode mechanism in a system at equilibrium is described by:

1 $${M}^{z+}(aq)+n{\rm{I}}(org)+z{{\rm{CH}}}^{+}(org)\leftrightarrow M{{\rm{I}}}_{n}^{z+}(org)+z{{\rm{C}}}^{-}(org)+z{{\rm{H}}}^{+}(aq)$$

where, *M*, *I*, and *C* are the ion of interest, ionophore, and chromoionophore, respectively, and *aq* and *org* denote an ion, *M*, in aqueous or organic phase of the optode component, respectively. The exchange constant to describe the partitioning between the two phases in the ion-exchange system is then given by:

2 $${K}_{exch}^{M{{\rm{I}}}_{n}}={(\frac{{\alpha }_{H}[{\rm{C}}]}{[{{\rm{CH}}}^{+}]})}^{z}\frac{[M{{\rm{I}}}_{n}^{z+}]}{{\alpha }_{M}{[{\rm{I}}]}^{n}}={(\frac{{K}_{\alpha }}{{K}_{H}})}^{z}{k}_{{\rm{M}}}{\beta }_{{\rm{M}}{I}_{n}}$$

where, α_H_ and α_M_ denote the activity of a proton, *H*, or the ion, *M*, in the sensor. The optical activity reflects the concentration of respective complexes within the organic phase of the optode component by maintaining electroneutrality^46^. Under these conditions, the relation between the deprotonation ratio (α) of the nanosensor and the ion activity is:

3 $${\alpha }_{{\rm{I}}}={(z{K}_{exch}^{M{{\rm{I}}}_{n}})}^{-1}{(\frac{1-\alpha }{\alpha }{\alpha }_{H})}^{z}\times \frac{{R}_{T}^{-}-\alpha {C}_{T}}{{\{{I}_{T}-({R}_{T}^{-}-\alpha {C}_{T})(n/z)\}}^{n}}$$

For sensor calibration, the emission intensity ratios of two fluorophores (CHIII:R18, Eqs 4–6, see Methods) in response to Ca^2+^ was used to plot a calibration curve with a Hill fit to determine effective Ca^2+^ concentration at half-maximal sensor response (defined here as EC_50_). The emission spectra for individual fluorophores are shown in Supplementary Figure S1. The sensing range can be fine-tuned (Fig. 2b) by altering the amount of the individual sensing components (CaI-II, CHIII, NaTFPB, see Methods) as previously demonstrated^53^.

For intracellular applications, the ratiometric response of nanosensor can be tuned to exhibit EC_50_ at 105 ± 19 nM in solution (Fig. 2c), which is close to 100 nM intracellular calcium basal level^48^. There were batch-to-batch differences in sensor response and calibrations were routinely performed to ensure the sensors maintain optimal responsiveness for subsequent cellular studies. An index of nanosensor sensitivity, as determined by the slope of linear portion of the normalized response at EC_50_ (Fig. 2c, inset), measured a decrease of 80% between 38 to 600 nM.

For sensor selectivity, the sensor responded to Ca^2+^ with high selectivity over the primary intracellular interfering ion, magnesium (Mg^2+^) (Fig. 2d). The opCaNS demonstrated EC_50_ for magnesium >1 mM, which produces a log selectivity coefficient of 4 (Eq. 7, zero background ion). The physiological range of ionized Mg^2+^ concentration is known to be 0.5–1 mM^54^, suggesting there is minimal interference of sensor response by Mg^2+^.

It is critical to determine the sensor response time to assess the ability of nanosensors to capture ionic transients which occur on the millisecond-to-second time scale^47–50^. Response time of the nanosensor was assessed by a two-photon (2P) Ca^2+^ uncaging experiment (Fig. 2e). We first demonstrated that the opCaNS is 2P-excitable with maximum fluorescence observed at excitation wavelengths centered at 875 nm (Supplementary Figure S2). Subsequent uncaging data shows that nanosensors responded instantaneously to elevations of local Ca^2+^ levels when imaged at a frame-rate of 6 Hz, as indicated by an increase of fluorescence from baseline. This suggests the capability of nanosensors to capture intracellular Ca^2+^ signals by store-release mechanisms (see next section). It is worth noting that the measured response time of the nanosensors here is limited by the imaging frame-rate, and the temporal resolution can be significantly improved by other imaging methods^31^.

Reversibility is also key to monitoring Ca^2+^ release from the intracellular store due to its rise and decay characteristics^47–50^. The ratiometric response of the opCaNS exhibited a clear reversibility profile (Fig. 2f) over repeated cycles of alternating Ca^2+^ concentrations (100 and 1350 nM), which is indicative of excellent cycling capability of the nanosensors. The reversibility profile for individual fluorophores (CHIII and R18) is shown in Supplementary Figure S3.

Collectively, the results show applicable sensor characteristics for reporting local cytoplasmic Ca^2+^ concentration by exhibiting nanomolar response range, high selectivity, fast response time, and reversible response.

### Sensing intracellular calcium signals

**Figure 3:**
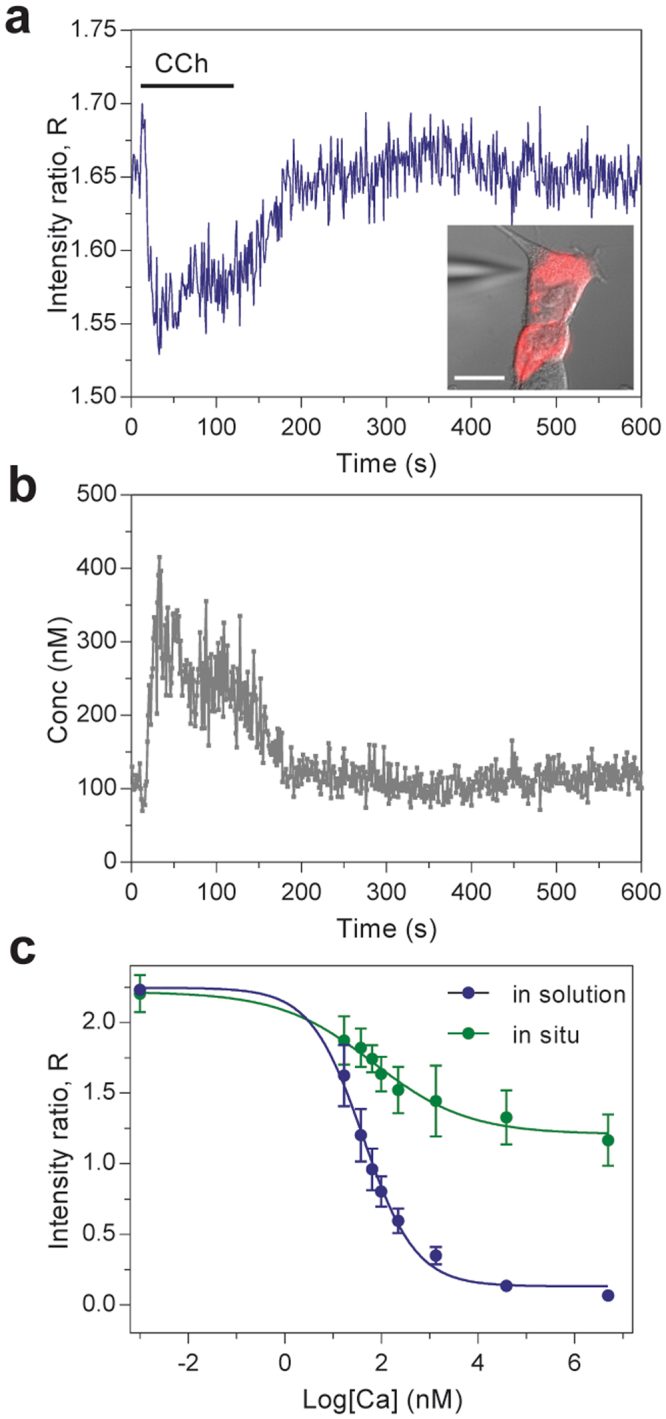
Intracellular Ca^2+^ detection by carbachol-mediated calcium store release. (**a**) Time-lapse confocal imaging of nanosensor-loaded cells (inset) reveal an initial decrease in fluorescence intensity ratio followed by an increase to the base level, indicating a rise in intracellular Ca^2+^ level followed by cytoplasmic depletion. (**b**) Conversion of fluorescence signal to Ca^2+^ levels indicate cytosolic elevations reach up to ~400 nM. (**c**) *In situ* cell calibration (green; EC_50_ = 110 ± 42 nM) was performed by superfusing varying concentrations of Ca^2+^ in presence of ionomycin to allow Ca^2+^ levels to be equilibrated across the cell membrane. This calibration is compared with the result (EC_50_ = 54 ± 14 nM) obtained from in-solution calibration experiment using identical nanosensors (blue). CCh: carbachol. Scale bar: 10 µm.

To demonstrate the capabilities of our nanosensors for intracellular applications, we examined carbachol-induced Ca^2+^ store release in HeLa cells (*n* = 3) which acts on inositol 1,4,5-triphosphate receptors (IP_3_Rs) on the endoplasmic reticulum (ER) membrane^55–57^. First, nanosensors were loaded into HeLa cells using pressure controlled microinjection (Fig. 3a, inset). Although nanosensors have previously been introduced to intracellular space through endocytosis^39, 58^, the microinjection technique ensures nanoparticle delivery to the cell cytosol and prevents encapsulation by endosomes^31, 59–61^. Each injection was about one tenth of total volume correlating to ~10^2^−10^3^ nanosensors (picomolar) per cell (Supplementary Figure S4), which is an amount that provided robust signal-to-noise ratio for imaging without inflicting damage to the cells.

Time-lapse confocal imaging (Fig. 3a) revealed an initial short-lived decrease followed by a slower increase in sensor intensity ratio. This indicates an elevation of Ca^2+^ levels followed by a slow decay of cytoplasmic Ca^2+^ to its basal level. When the intensity ratios are converted to Ca^2+^ concentration, the cytosolic elevations reached up to ~400 nM (Fig. 3b). The empirical conversion involved performing *in situ* cell calibration (Fig. 3c) and curve fitted by the Hill equation (Eq. 8) to determine the EC_50_ after the carbachol experiment on the same cell. Briefly, *in situ* cell calibrations were performed by superfusing nanosensor-loaded HeLa cells with varying concentrations of Ca^2+^ using calibration buffer in presence of 5 µM ionomycin, which serve as ion carriers to facilitate the equilibrium of Ca^2+^ levels across the cell membrane. Although measurement of EC_50_ in solution can serve as an index of sensor performance, the behavior can change when loaded in living cells^62, 63^. As shown in Fig. 3c, the EC_50_ of *in situ* cell calibration was determined to be 110 ± 42 nM. In comparison, the EC_50_ of the same batch of nanosensor in solution was 54 ± 14 nM. We note that the differences between *in situ* and in solution calibrations in our sensor response (EC_50_) is less or comparable to those observed in previous studies using dye indicators and PEBBLE NP-sensors (Table S1), suggesting the validity of nanosensors for intracellular studies. The *in situ* calibration curve defined by optode function, α, is also provided in Supplementary Figure S5. The recovery of Ca^2+^ signal to baseline in our sensor carbachol experiment suggests that reversible characteristics of the nanosensors remained intact. Also, comparable signal amplitudes were observed from Ca^2+^ indicator, Fluo-4^64^ (Supplementary Figure S6), suggesting that taken together, we demonstrate the use of optode-based nanosensors as a sensing platform for monitoring and quantitative imaging of intracellular ion dynamics.

## Discussion

The main advantage of our sensor platform is the flexibility to tune the sensor to a given response range and selectivity for various applications by altering the sensing components. While molecular probes can improve individual aspects of indicator performance such as Kd, wavelength, and ratiometric measurements, NP-based sensors benefit from addressing these issues simultaneously. For example, sensor sensitivity can be tuned to a dynamic range of interest which can mitigate issues with indicator buffering and affinities that are either lower or higher relative to the magnitude of the Ca^2+^ signal - which often leads to indicator saturation and causing erroneous estimation of the magnitude of the signal^65, 66^. Also, the current sensing scheme utilizes a recognition agent that is separate from an optical signal transducer. As a result, a wide range of highly selective ionophores^22, 31, 32^ applied for potentiometric biosensing^51, 67–72^ can be explored for optical detection of other physiologically important ions that lack such specific probes. In addition, the current nanosensor platform permits selection of fluorophores in the visible and infrared range to permit ratiometric imaging where possible photodamaging effects induced by UV light excitation of common ratiometric indicators such as Fura-2^2, 13^ and Indo-1^14^, can be minimized. While the damage imposed on cells was reduced during microinjection procedure where possible, microinjection is an invasive method which may perturb the normal physiology and delay restoration of elevated Ca^2+^ levels. This can be improved by further sensor miniaturization through bottom-up fabrication approaches^22, 73^ and sensor delivery through facilitated uptake methods such as functionalizing nanosensors to cell penetrating peptides (CPPs)^73–75^ in order to minimize physical perturbation of living cells. Lastly, we find that the nanosensors are dynamically fluorescent under 2P excitation, demonstrating their potential use *in vivo*.

Similar to the pH dependent nature of conventional dye indicators^76, 77^, our nanosensor response is also subject to pH changes due to the encapsulation of pH-sensitive dyes. This is illustrated in Supplementary Figure S7, based on theoretical optode function (Eq. 3). However, the intracellular environment is governed and protected by a buffered system to maintain pH homeostasis near pH 7.2^78^, and the effect of pH is minimized. Also, the CHIII (ETH 5350) used in this work has a pKa of >10^21^, which is highly protonated under standard physiological conditions, and as such, the predominant sensor response is dictated by the changes in Ca^2+^ concentrations. Currently, there are developments of other efforts to circumvent such pH dependency^52, 79^.

This work expands on the continual development of optode-based nanosensors and highlights its importance as tools for monitoring physiological analytes. The current work introduces a ratiometric method for demonstrating quantitative determination of intracellular Ca^2+^ dynamics using these sensors which has not been described previously. The current sensor platform combines the ease of synthesis and the flexibility to allow incorporation of different sensing components. As such, the sensor response can be tuned for specific dynamic range, selectivity, and wavelengths. In future work, detection of other physiologically important ions such as sodium, potassium, and chloride will be explored for intracellular signaling applications.

## Methods

### Reagents

Bis(2-ethylhexyl) sebacate (DOS), calcium ionophore II (CaI II; *N,N,N′,N′*-Tetra[cyclohexyl]diglycolic acid diamide), chromoionophore III (CHIII; 9-(Diethylamino)-5-[(2-octyldecyl)imino]benzo[a]phenoxazine, ETH 5350), dichloromethane (DCM), ethylene glycol tetraacetic acid (EGTA), 4-(2-Hydroxyethyl)piperazine-1-ethanesulfonic acid (HEPES), magnesium chloride (MgCl_2_), sodium tetrakis-[3,5-bis(trifluoromethyl)phenyl]-borate (NaTFPB), and tetrahydrofuran (THF) were obtained from Sigma-Aldrich. 1-(4,5-dimethoxy-2-nitrophenyl)-1,2-diaminoethane-*N,N,N′,N′*-tetraacetic acid (DMNP-EDTA), octadecyl rhodamine B chloride (R18), ionomycin, calcium calibration buffer kit (C3008MP), Fluo-4 AM, and all cell culture media and supplements were obtained from Thermo Fisher. Tris-base was obtained from Fisher BioReagents. 1,2-distearoyl-*sn*-glycero-3-phosphoethanolamine-N-[methoxy(polyethylene glycol)-550] (ammonium salt) (DSPE-mPEG550) was obtained from Avanti Polar Lipids, Inc.

### Instrumentation

Fluorescent spectra for routine nanosensor calibration in solution and selectivity experiments were measured with Spectramax M3 plate reader (Molecular Devices). Sensor reversibility test and cell imaging were performed with Zeiss LSM700 inverted confocal laser scanning microscope (Carl Zeiss). Hydrodynamic diameter and zeta-potential measurements were determined using a 90 Plus particle size analyzer (Brookhaven Instruments Corporation). Ca^2+^ uncaging/imaging experiments were carried out on a two-photon microscope (Bruker) equipped with two Ti:Sa lasers (SpectraPhysics) and separate beam paths/galvanometer mirrors for independent imaging and uncaging. Laser power was controlled independently for each beam path using two Pockels cells (Conoptics).

### Nanosensor Fabrication

To prepare the opCaNS, the calcium-selective optode sensing components comprising CaI-II (2 μmol, 2 mg), CHIII (878 nmol, 0.5 mg), NaTFPB (1.12 μmol, 1 mg), and R18 (123 nmol, 0.09 mg) were first reconstituted separately in 300 μL THF. DOS (428 μmol, 200 μL) was then added to the optode sensing components and vortexed briefly for 30 s to form a homogeneous solution. Just prior to nanosensor fabrication, DSPE-mPEG550 (250 μg) was dried in a glass scintillation vial and rehydrated in HEPES buffer (130 mM KCl; 10 mM NaCl; 1 mM MgCl_2_, 10 mM HEPES; pH adjusted to 7.2 with tris base). In a typical opCaNS fabrication, 50 μL of polymer-free optode was added to 70 μL DCM, and this mixture was sonicated with the 4 mL of HEPES buffer containing DSPE-mPEG550 at 10% intensity for 1 min (Branson digital sonifier S-450D; 1/8′′ diameter tip). After sonication, the organic solvents were removed using a rotavap (Buhle) for 15 min at room temperature, and the resulting emulsion was filtered with a 100 nm syringe filter (Millipore).

### Particle Size and Zeta-Potential Measurement

Hydrodynamic diameters were measured by dynamic light scattering (DLS) based on the intensity of scattered 640 nm light at a fixed angle of 90°. All particle size measurements were taken in triplicates with sample solutions diluted in 10 mM HEPES buffer, pH 7.2 adjusted to a detector count rate of 150–450 kcps. Zeta-potentials were measured in PALS mode collected over 25 cycles each 10 runs per cycle. Size and zeta-potential measurements were analyzed from three separate batches of nanosensors each from three discrete optode stock preparations.

### Fluorescent Response to Calcium

Nanosensors were calibrated in solution for fluorescent response to a series of Ca^2+^ EGTA buffers with controlled concentrations of free calcium ion (Ca^2+^ calibration buffer). In order to determine the fraction protonated during measurements in Ca^2+^, acid and base standards were used as endpoints for fully protonated or deprotonated chromoionophore conditions, respectively.

The fluorescence intensities for CHIII (λ_EX_: 639 nm, λ_EM_: 670 nm) and R18 (λ_EX_: 555 nm, λ_EM_: 575 nm) were measured with plate reader in bottom read mode through clear-bottom 96-well plates (emission spectra of the fluorophores shown in Figure S1). The emission fluorescence intensity ratio, *R*, of two fluorophores (CHIII:R18) was calculated as:

4 $$R=\frac{Emission\,at\,670\,nm}{Emission\,at\,575\,nm}$$

Traditionally, optode data is converted to a normalized value termed α, by ratios of 670 nm/575 nm defined as:

5 $$\alpha =\frac{R-{R}_{D}}{{R}_{P}-{R}_{D}}$$

where, *R* is the fluorescence ratio measured experimentally, and *R*_*P*_ and *R*_*D*_ is the ratio of fully protonated and deprotonated state of the chromoionophore, respectively. The effective Ca^2+^ concentration at half-maximal sensor response (defined here as, EC_50_), for the nanosensors was calculated from Ca^2+^ concentrations and the corresponding values of alpha, α, was fitted according to the dose-response (Hill) equation:

6 $$R(\alpha )={R}_{\min }+\frac{{R}_{\max }-{R}_{\min }}{1+{10}^{(LogE{C}_{50}-LogC)p}}$$

where *R*(α) is intensity ratio of measured response, *C*, denotes Ca^2+^ concentration at which a ratio was measured, and *R*_min_, *R*_max_, and EC_50_ are parameters obtained from the fitted sigmoidal curve defined by the Hill equation.

### Selectivity for Calcium over Magnesium

Nanosensor selectivity was determined by measuring the ratiometric fluorescent response (CHIII:R18) to Ca^2+^ and Mg^2+^ as described above, and fitting the calibration data to the equation comparable to the Nicolskii-Eisenman model with a fixed interfering ion^51, 80^. The selectivity coefficient for an optical sensor is defined as:

7 $$Log{K}_{ij}^{opt}=LogE{C}_{50}^{i}-LogE{C}_{50}^{j}$$

where, $E{C}_{50}^{i}$ and $E{C}_{50}^{j}$ are the constants for the interfering ion (Mg^2+^) and primary ion (Ca^2+^), respectively.

### Confocal Microscope Settings

For reversibility and cell imaging experiments (see below), confocal laser scanning microscope (Zeiss LSM 700, Carl Zeiss) was used to measure fluorescence intensity ratios (CHIII:R18), as described above. Briefly, simultaneous images were acquired from dual excitation (λ_EX_: 555 and 639 nm) and peak intensities were recorded at two emission channels (R18 λ_EM_: 575 nm and CHII λ_EM_: 670 nm) using diffraction grating set to split the emission at 620 nm. The 40× oil-immersion objective lens (NA = 1.3) was used with the pinhole aperture setting at 2 µm which remained constant throughout the entire experiment.

### Sensor Reversibility

For reversibility experiments, nanosensors were firstly bound to the surface of a neutravidin functionalized rectangular glass flowchamber (0.1× 2 mm ID) as described by Reinhard *et al*.^81^, and imaged on the confocal microscope. Simultaneous images from both color channels were acquired every 10 s intervals with alternating solutions of 100 or 1350 nM calcium buffers exchanged every 2 min for five exchange cycles. Images were analyzed using Matlab with identical regions of interest defined on both channels.

### Two-Photon Uncaging and Imaging

Calcium caging agent (DMNP, 1.05 mM) and opCaNS were loaded into 20 μL glass capillaries (Drummond) with 1 mM Ca^2+^, and the ends of the capillary were sealed with wax. Imaging was carried out at 875 nm excitation wavelength through a 20 ×/0.45 N.A. objective (Nikon). Images were collected via photomultiplier tube (PMT, Hamamatsu) after a 595/50 emission filter (Semrock), at a frame-rate of 6 Hz. For DMNP uncaging, the uncaging beam (730 nm) dwelled on the target for 500 ms. Images were analyzed using ImageJ to extract raw data at the site of uncaging pulse (20 × 20 pixels).

### Cell Culture

HeLa cells (Sigma, 93021013) were grown in Eagle’s Minimum Essential Medium (EMEM) supplemented with 10% fetal bovine serum, 2 mM glutamine, 1% non-essential amino acids (NEAA), penicillin (50 U/ml), and streptomycin (50 μg/ml) at 37 °C in a humidified atmosphere containing 5% CO_2_. The cells were plated on poly-d-lysine coated 50 mm glass bottom dishes at a density of 4–8 × 10^4^ cells. For Ca^2+^ imaging studies, cells were cultured in serum-free medium for 12 h, washed with PBS, and subsequently imaged using Ca^2+^-free Hank’s Balanced Salt Solution (HBSS; Thermo Fisher, 14175-079) with 10 mM HEPES, adjusted to pH 7.2 (Ca^2+^-free imaging buffer).

### Delivery and *in situ* Cell Calibration

Nanosensors were delivered to cytosolic compartment of HeLa cells using a microinjection technique (Eppendorf InjectMen 4) with 1.0 mm/0.5 mm (OD/ID) borosilicate glass tubes pulled to a tip size 0.1–0.5 µm using P-97 micropipette puller (Sutter Instrument). For *in situ* cell calibration, nanosensor loaded cells were initially incubated with Ca^2+^-free imaging buffer containing 5 µM ionomycin for 15 min to equilibrate Ca^2+^ levels within the cytosolic environment to the extracellular space. Ca^2+^ calibration buffer with incremental levels of Ca^2+^ were subsequently added and fluorescence images from the confocal microscope were acquired after 10 min incubation at each calcium concentration. The values of alpha, α, and the EC_50_ was determined based on the same methods used to calibrate sensors in solution. There were batch-to-batch differences in sensor response and consequently, in solution and *in situ* cell calibrations were routinely performed to ensure the sensors maintain responsiveness to the intracellular Ca^2+^ levels.

### Carbachol-Induced Intracellular Calcium Measurement

Single cell determination of the intracellular calcium concentration ([Ca^2+^]_i_) was carried out in opCaNS-loaded HeLa cells. Carbachol (1 mM) was used to invoke changes in [Ca^2+^]_i_ by acting on IP_3_R on the endoplasmic reticulum (ER) membrane. Fluorescence confocal images were acquired at 1 s interval for 10 min after addition of carbachol to the 50 mm culture dish. Fluorescence intensities in the regions of interest (ROI) on each channel (575 nm and 670 nm) were first background subtracted by the intensities of nearby area that were void of cells. For individual regions of interest (ROI) in the cytosolic cellular region we subsequently used the corrected fluorescence intensities on both channels to calculate intensity ratio *R* (*F*_670_ /*F*_575_) normalized by the photobleaching profile. Changes in nanosensor fluorescence ratio were expressed as changes in Ca^2+^ concentration based on *in situ* calibrations as described above. Conversion of fluorescence ratio to Ca^2+^ concentration, *C*, involved empirically deriving from acquired fluorescence ratio using the dose-response (Hill) equation (Eq. 6) and plotted against time. Image processing and data analysis were performed using Matlab and Origin.

8 $$C=E{C}_{50}\sqrt[p]{\frac{R-{R}_{\min }}{{R}_{\max }-R}}$$

### Data and Image Analysis

Data and image analysis was performed using Matlab and images were constructed in Origin 8 (OriginLab). Numerical values are represented as a mean ± standard deviation, and graphical representations contain error bars for the standard deviation values.

## Electronic supplementary material

Supporting Information
