# Supplementary material for: A method for estimating intracellular ion concentration using optical nanosensors and ratiometric imaging
Source: Sci Rep. 2017 Sep 7;7:10819. doi: 10.1038/s41598-017-11162-8 (PMC5589868; doi:10.1038/s41598-017-11162-8)
Supplement: Supplementary file 1 — Supporting Information [file 41598_2017_11162_MOESM1_ESM.pdf]

Supporting Information for:

A method for estimating intracellular ion concentration using optical nanosensors and ratiometric imaging

Guoxin Rong,[1,2,5] Eric H. Kim,[1,2,5] Kira E. Poskanzer[4] and Heather A. Clark[2,3,*]

1. Department of Pharmaceutical Sciences, Northeastern University, Boston, MA 02115, United States

2. Department of Bioengineering, Northeastern University, Boston, MA 02115, United States

3. Department of Chemistry and Chemical Biology, Northeastern University, Boston, MA 02115, United States

4. Department of Biochemistry & Biophysics, University of California, San Francisco, CA 94143, United States

5. These authors contributed equally to this work.

Supplementary Table:

Table S1. Comparison of in vitro and in situ cell calibration values for different calcium indicators and nanosensors.

**Indicator / Nanosensor**	**K_d *in vitro* (nM)**	**K_d *in situ* (nM)**	**References**
Calcium Green-1	190	930	(1)
Fluo-4	345	1000	(1)
Fura-2	145	350	(2)
Rhod-2	570	720	(3)
PEBBLE (Rhod-2)	478	293	(4)
opCaNS	54 (EC_{50})	110 (EC_{50})	

Supplementary Figures:

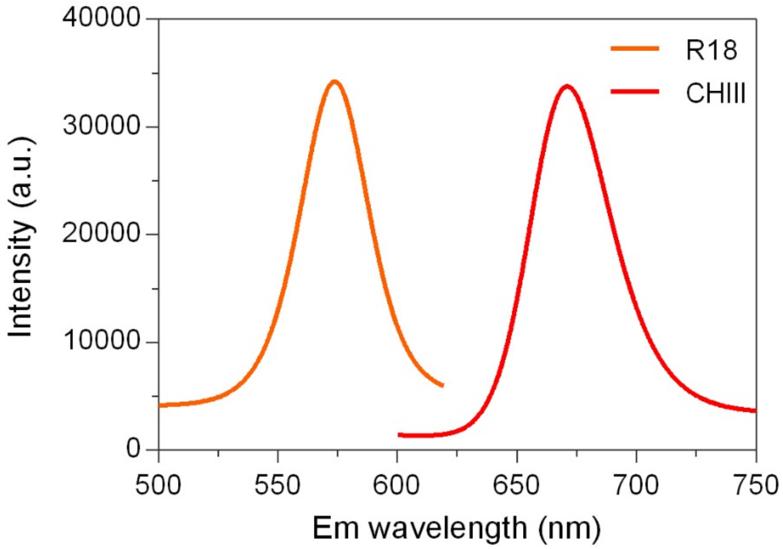

Figure S1. Emission spectra of individual fluorophores (R18 and CHIII) in the nanosensor construct. Peak emission intensity of R18 and CHIII is observed at 575 nm and 670 nm, respectively.

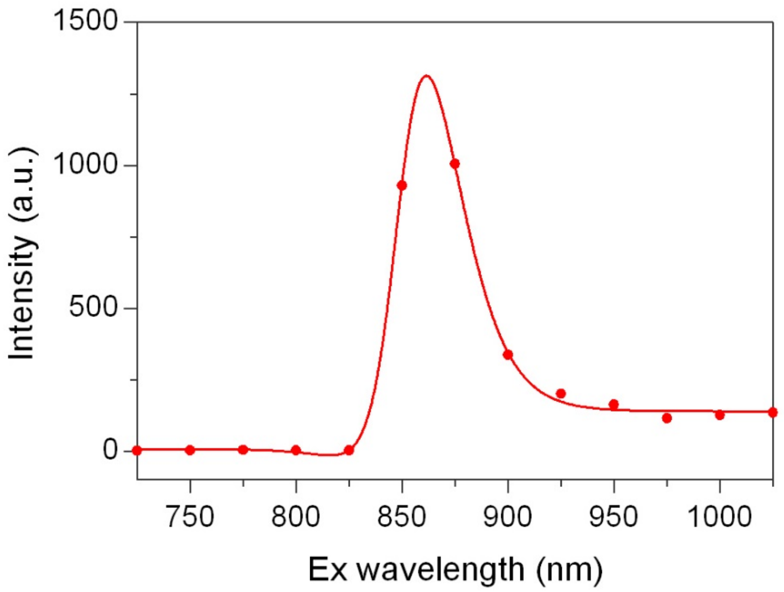

Figure S2. Two-photon excitation spectra of opCaNS. The excitation wavelength maxima occurs at 875 nm.

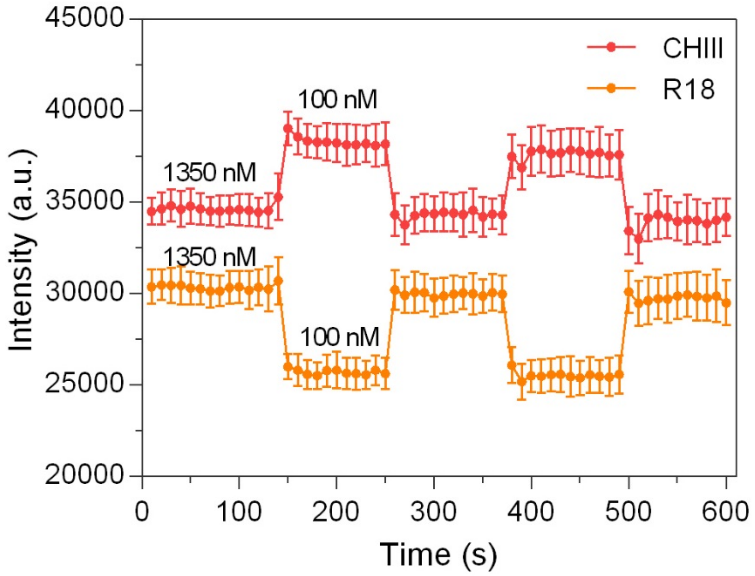

Figure S3. Reversibility profile of individual fluorophores (R18 and CHIII) showing opposite trend to varying calcium concentrations (100 and 1350 nM).

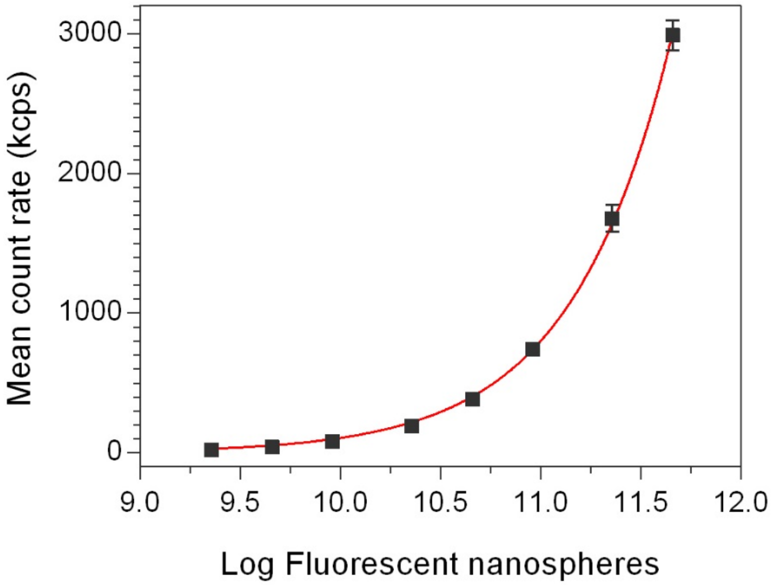

Figure S4. Determination of relationship between nanosensor concentration and count rates measured by dynamic light scattering (DLS). The mean count rates of known concentrations of polystyrene (PS) microspheres are plotted. Data points shown are the average count rates (n = 3) with increasing concentrations of PS microspheres. Error bars indicate one standard deviation. An exponential line is fitted to the data (red).

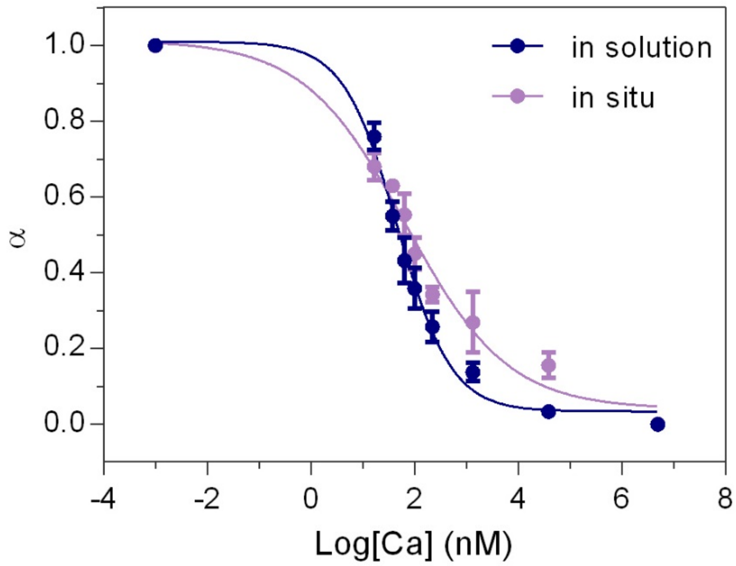

Figure S5. *In situ* cell calibration curve defined by optode function, α, in the cell (purple; EC_{50} = 70 ± 28 nM) in comparison to in-solution (blue; EC_{50} = 42 ± 6 nM) using identical nanosensors.

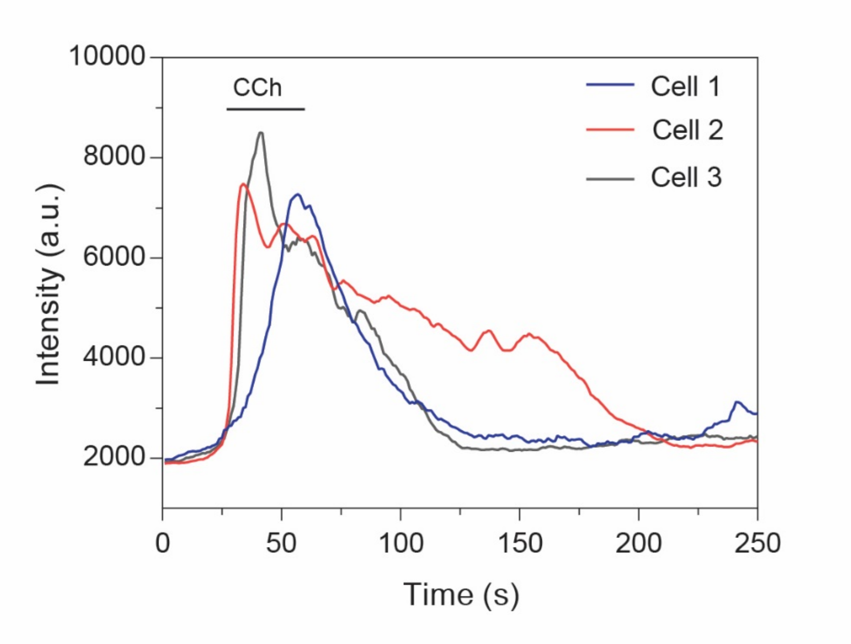

Figure S6. Carbachol induced intracellular Ca^{2+} fluorescence response using Fluo-4 in HeLa cells.

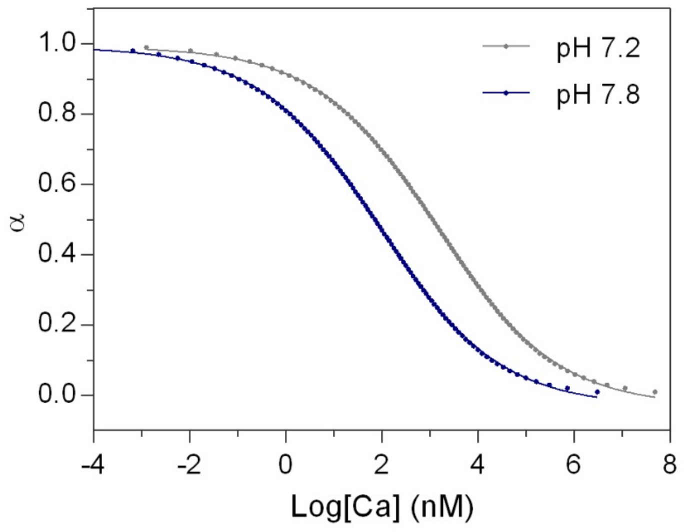

Figure S7. Theoretical pH effect of calcium ion-selective optode response simulated using **Eq. 3**.

References

(1) Thomas, D.; Tovey, S. C.; Collins, T. J.; Bootman, M. D.; Berridge, M. J.; Lipp, P. *Cell Calcium* **2000**, *28*, 213.
(2) Negulescu, P. A.; Machen, T. E. *Methods Enzymol* **1990**, *192*, 38.
(3) Du, C.; MacGowan, G. A.; Farkas, D. L.; Koretsky, A. P. *Cell Calcium* **2001**, *29*, 217.
(4) Si, D.; Epstein, T.; Lee, Y. E.; Kopelman, R. *Anal Chem* **2012**, *84*, 978.
